# Adamantane-1-ammonium acetate

**DOI:** 10.1107/S1600536811012670

**Published:** 2011-05-07

**Authors:** Elise J. C. de Vries, Caryn Gamble, Monika Nowakowska

**Affiliations:** a Molecular Science Institute, School of Chemistry, University of the Witwatersrand, PO Wits, 2050 Johannesburg, South Africa

## Abstract

In the title compound, C_10_H_18_N^+^·C_2_H_3_O_2_^−^, the ammonium H atoms of the cation are linked to three acetate anions *via* N—H⋯O hydrogen bonds, forming a chain structure extending along the *b* axis.

## Related literature

For related structures, see: Mullica *et al.* (1999 ▶); He & Wen (2006 ▶). For their applications in virology, see: Hoffmann (1973) ▶; Dolin *et al.* (1982 ▶); Bright *et al.* (2005 ▶); Betakova (2007 ▶). For graph-set analysis, see: Bernstein *et al.* (1995 ▶). For C*sp* ^3^—O bond lengths, see: Orpen *et al.* (1989 ▶). 
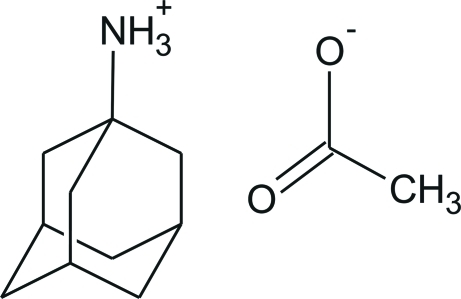


## Experimental

### 

#### Crystal data

C_10_H_18_N^+^·C_2_H_3_O_2_^−^
*M* *_r_* = 211.30
Monoclinic, 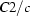

*a* = 25.7625 (12) Å
*b* = 6.4852 (3) Å
*c* = 17.3970 (9) Å
β = 127.377 (2)°
*V* = 2309.8 (2) Å^3^
*Z* = 8
Mo *K*α radiation
μ = 0.08 mm^−1^
*T* = 296 K
0.40 × 0.07 × 0.05 mm

#### Data collection

Bruker APEXII CCD diffractometer
15067 measured reflections
2261 independent reflections
1471 reflections with *I* > 2σ(*I*)
*R*_int_ = 0.077

#### Refinement

*R*[*F* ^2^ > 2σ(*F* ^2^)] = 0.052
*wR*(*F* ^2^) = 0.134
*S* = 1.01
2261 reflections
149 parameters
H atoms treated by a mixture of independent and constrained refinement
Δρ_max_ = 0.23 e Å^−3^
Δρ_min_ = −0.21 e Å^−3^

### 

Data collection: *APEX2* (Bruker, 2005 ▶); cell refinement: *SAINT-NT* (Bruker, 2005 ▶); data reduction: *SAINT-NT*; program(s) used to solve structure: *SHELXS97* (Sheldrick, 2008 ▶); program(s) used to refine structure: *SHELXL97* (Sheldrick, 2008 ▶); molecular graphics: *X-SEED* (Barbour, 2001 ▶; Atwood & Barbour, 2003 ▶); software used to prepare material for publication: *X-SEED*.

## Supplementary Material

Crystal structure: contains datablocks global, I. DOI: 10.1107/S1600536811012670/zs2106sup1.cif

Structure factors: contains datablocks I. DOI: 10.1107/S1600536811012670/zs2106Isup2.hkl

Additional supplementary materials:  crystallographic information; 3D view; checkCIF report

## Figures and Tables

**Table 1:** Hydrogen-bond geometry (Å, °)

*D*—H⋯*A*	*D*—H	H⋯*A*	*D*⋯*A*	*D*—H⋯*A*
N1—H1*A*⋯O1	0.99 (4)	1.80 (4)	2.777 (3)	171 (3)
N1—H1*B*⋯O2^i^	0.94 (3)	1.83 (3)	2.758 (3)	170 (3)
N1—H1*C*⋯O1^ii^	0.97 (2)	1.82 (2)	2.786 (2)	171 (3)

Symmetry codes: (i) 
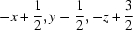
; (ii) 
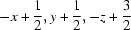
.

**Fig. 1.**
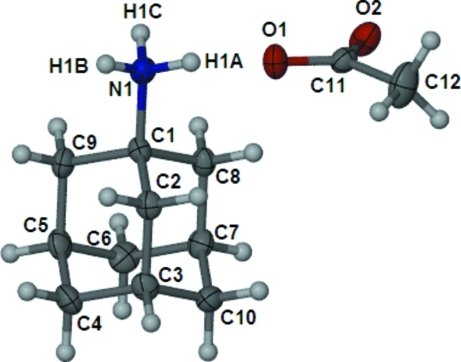
The atom numbering scheme of compound (I). Displacement ellipsoids are drawn at 50% probability level.

**Fig. 2.**
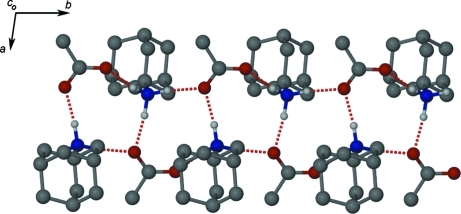
A perspective diagram viewed down the c axis of the molecular packing arrangement in (I), displaying the N–H···O hydrogen-bonding contacts as red dashed lines. Hydrogen atoms not involved in hydrogen bonding are omitted.

## supplementary crystallographic information

### Comment

It is well established that 1-aminoadamantane hydrochloride (amantadine hydrochloride: trade name Symmetrel) is effective in the prevention and treatment of the influenza (A) virus (Hoffmann, 1973; Dolin *et al.*, 1982; Bright *et al.*, 2005). However recent studies suggest that the virus is becoming increasingly resistant to this anti-influenza drug (Betakova, 2007). The investigation of new derivatives of this compound is still important. Here we report the crystal structure of adamantane-1-ammonium acetate (I), illustrated in Fig. 1.

The asymmetric unit of (I) contains an adamantane-1-ammonium cation and an acetate anion. In the cation, the exocyclic C—N bond is 1.500 (3) Å. The C—C bonds of the adamantane skeleton range from 1.527 (3) to 1.537 (3) Å, with a mean value of 1.531 Å. The C—C—C bond angles range from 109.05 (18) to 110.10 (18) °, with a mean value of 109.5 °, which is in good agreement with the value for a tetrahedral angle. In the anion, the C(sp3)-O distances of 1.266 (3) and 1.240 (3) Å are in the range of values given in the literature (Orpen *et al.*, 1989).

The crystal structure is stabilized by a network of intermolecular charge assisted carbonyl-to-amine hydrogen bonds. All ammonium hydrogen atoms are involved in hydrogen bonding with the oxygen atoms of the acetate anion (Table 1). The hydrogen-bonding scheme can be described as a nine membered ring motif with graph-set notation R^3^_4_(10) (Bernstein *et al.*, 1995). This results in the formation of a one-dimensional chain structure parallel to the *b* axis of the unit cell (Fig. 2).

### Experimental

Adamantadine hydrochloride (10 mg) was dissolved in three drops of glacial acetic acid and deionized water. The solution was allowed to undergo slow evaporation. Single crystals of (I) suitable for X-ray diffraction anlaysis precipitated after a few days.

### Refinement

The ammonium H atoms were placed according to the observed electron density and allowed to refine freely. A distance constraint was placed on one of the N—H bonds [N1–H1C, 0.95 (2) Å]. The remaining H atoms were positioned geometrically and allowed to ride on their respective parent atoms, with C—H bond lengths of 0.99 (aromatic CH) 1.00 (methine CH), 0.99 (methylene CH_2_) and 0.98 Å (methyl CH_3_), and with *U*_iso_(H) = 1.2 or 1.5 times *U*_eq_(C).

### Crystal data

**Table:**

C_10_H_18_N^+^·C_2_H_3_O_2_^−^	*F*(000) = 928
*M**_r_* = 211.30	*D*_x_ = 1.215 Mg m^−^^3^
Monoclinic, *C*2/*c*	Mo *K*α radiation, λ = 0.71073 Å
Hall symbol: -C 2yc	Cell parameters from 1706 reflections
*a* = 25.7625 (12) Å	θ = 3.0–25.1°
*b* = 6.4852 (3) Å	µ = 0.08 mm^−^^1^
*c* = 17.3970 (9) Å	*T* = 296 K
β = 127.377 (2)°	Needle, colourless
*V* = 2309.8 (2) Å^3^	0.40 × 0.07 × 0.05 mm
*Z* = 8	

### Data collection

**Table:**

Bruker APEXII CCD diffractometer	1471 reflections with *I* > 2σ(*I*)
Radiation source: fine-focus sealed tube	*R*_int_ = 0.077
graphite	θ_max_ = 26.0°, θ_min_ = 2.0°
φ and ω scans	*h* = −31→31
15067 measured reflections	*k* = −8→8
2261 independent reflections	*l* = −21→20

### Refinement

**Table:**

Refinement on *F*^2^	Primary atom site location: structure-invariant direct methods
Least-squares matrix: full	Secondary atom site location: difference Fourier map
*R*[*F*^2^ > 2σ(*F*^2^)] = 0.052	Hydrogen site location: inferred from neighbouring sites
*wR*(*F*^2^) = 0.134	H atoms treated by a mixture of independent and constrained refinement
*S* = 1.01	*w* = 1/[σ^2^(*F*_o_^2^) + (0.0546*P*)^2^ + 1.9569*P*] where *P* = (*F*_o_^2^ + 2*F*_c_^2^)/3
2261 reflections	(Δ/σ)_max_ < 0.001
149 parameters	Δρ_max_ = 0.23 e Å^−^^3^
0 restraints	Δρ_min_ = −0.21 e Å^−^^3^

### Special details

**Table:**

Geometry. All e.s.d.'s (except the e.s.d. in the dihedral angle between two l.s. planes) are estimated using the full covariance matrix. The cell e.s.d.'s are taken into account individually in the estimation of e.s.d.'s in distances, angles and torsion angles; correlations between e.s.d.'s in cell parameters are only used when they are defined by crystal symmetry. An approximate (isotropic) treatment of cell e.s.d.'s is used for estimating e.s.d.'s involving l.s. planes.
Refinement. Refinement of *F*^2^ against ALL reflections. The weighted *R*-factor *wR* and goodness of fit *S* are based on *F*^2^, conventional *R*-factors *R* are based on *F*, with *F* set to zero for negative *F*^2^. The threshold expression of *F*^2^ > σ(*F*^2^) is used only for calculating *R*-factors(gt) *etc*. and is not relevant to the choice of reflections for refinement. *R*-factors based on *F*^2^ are statistically about twice as large as those based on *F*, and *R*- factors based on ALL data will be even larger.

### Fractional atomic coordinates and isotropic or equivalent isotropic displacement parameters (Å^2^)

**Table:**

	*x*	*y*	*z*	*U*_iso_*/*U*_eq_	
O1	0.32323 (7)	0.4743 (2)	0.78287 (11)	0.0299 (4)	
N1	0.19171 (10)	0.5670 (3)	0.68283 (14)	0.0240 (5)	
C1	0.16138 (10)	0.5443 (3)	0.57747 (15)	0.0218 (5)	
O2	0.36698 (8)	0.7678 (2)	0.78092 (12)	0.0347 (4)	
C2	0.17908 (11)	0.3319 (3)	0.56130 (15)	0.0252 (5)	
H2B	0.2271	0.3184	0.6007	0.030*	
H2A	0.1628	0.2229	0.5815	0.030*	
C3	0.14826 (10)	0.3069 (3)	0.45404 (15)	0.0264 (5)	
H3A	0.1595	0.1679	0.4430	0.032*	
C4	0.07403 (11)	0.3266 (4)	0.39419 (17)	0.0327 (6)	
H4A	0.0570	0.2184	0.4137	0.039*	
H4B	0.0537	0.3075	0.3248	0.039*	
C5	0.05648 (11)	0.5400 (4)	0.41012 (17)	0.0299 (6)	
H5A	0.0079	0.5534	0.3705	0.036*	
C6	0.08313 (11)	0.7061 (4)	0.38012 (17)	0.0315 (6)	
H6A	0.0715	0.8443	0.3896	0.038*	
H6B	0.0633	0.6903	0.3107	0.038*	
C7	0.15743 (11)	0.6867 (3)	0.44084 (16)	0.0270 (5)	
H7A	0.1746	0.7962	0.4213	0.032*	
C8	0.18804 (10)	0.7119 (3)	0.54849 (15)	0.0243 (5)	
H8A	0.2362	0.7003	0.5881	0.029*	
H8B	0.1773	0.8496	0.5598	0.029*	
C9	0.08731 (10)	0.5654 (4)	0.51785 (16)	0.0267 (5)	
H9A	0.0761	0.7024	0.5291	0.032*	
H9B	0.0701	0.4585	0.5376	0.032*	
C10	0.17474 (11)	0.4742 (3)	0.42404 (17)	0.0287 (6)	
H10A	0.2227	0.4608	0.4626	0.034*	
H10B	0.1554	0.4578	0.3549	0.034*	
C11	0.36787 (11)	0.5780 (3)	0.79083 (16)	0.0251 (5)	
C12	0.42684 (13)	0.4608 (4)	0.8153 (2)	0.0465 (7)	
H12A	0.4638	0.4855	0.8832	0.070*	
H12B	0.4381	0.5079	0.7736	0.070*	
H12C	0.4169	0.3130	0.8052	0.070*	
H1A	0.2392 (15)	0.544 (4)	0.723 (2)	0.055 (8)*	
H1B	0.1765 (12)	0.461 (4)	0.7010 (17)	0.037 (7)*	
H1C	0.1835 (11)	0.704 (3)	0.6961 (17)	0.043 (7)*	

### Atomic displacement parameters (Å^2^)

**Table:**

	*U*^11^	*U*^22^	*U*^33^	*U*^12^	*U*^13^	*U*^23^
O1	0.0225 (9)	0.0286 (9)	0.0383 (10)	0.0000 (7)	0.0183 (8)	0.0033 (7)
N1	0.0244 (11)	0.0242 (11)	0.0265 (10)	0.0001 (9)	0.0170 (10)	0.0004 (9)
C1	0.0183 (11)	0.0227 (11)	0.0218 (11)	−0.0014 (9)	0.0109 (10)	0.0002 (9)
O2	0.0440 (11)	0.0258 (9)	0.0491 (11)	0.0017 (8)	0.0360 (9)	0.0018 (8)
C2	0.0240 (12)	0.0228 (11)	0.0282 (12)	0.0003 (10)	0.0155 (11)	0.0017 (10)
C3	0.0261 (13)	0.0210 (12)	0.0313 (13)	0.0004 (10)	0.0170 (11)	−0.0029 (10)
C4	0.0267 (13)	0.0352 (13)	0.0307 (13)	−0.0096 (11)	0.0145 (11)	−0.0073 (11)
C5	0.0171 (12)	0.0380 (14)	0.0286 (12)	−0.0004 (10)	0.0108 (10)	0.0007 (11)
C6	0.0287 (14)	0.0342 (14)	0.0277 (12)	0.0070 (11)	0.0151 (11)	0.0067 (11)
C7	0.0289 (13)	0.0265 (12)	0.0307 (13)	−0.0009 (10)	0.0208 (11)	0.0036 (10)
C8	0.0228 (12)	0.0206 (11)	0.0312 (12)	−0.0017 (9)	0.0173 (11)	−0.0015 (9)
C9	0.0218 (12)	0.0295 (13)	0.0326 (13)	0.0001 (10)	0.0185 (11)	0.0009 (10)
C10	0.0260 (13)	0.0354 (14)	0.0269 (12)	−0.0003 (10)	0.0171 (11)	−0.0038 (10)
C11	0.0258 (13)	0.0276 (12)	0.0255 (12)	0.0022 (10)	0.0174 (11)	0.0001 (10)
C12	0.0401 (16)	0.0417 (16)	0.072 (2)	0.0117 (13)	0.0416 (16)	0.0143 (14)

### Geometric parameters (Å, °)

**Table:**

O1—C11	1.265 (3)	C5—C9	1.537 (3)
N1—C1	1.501 (3)	C5—H5A	1.0000
N1—H1A	0.98 (3)	C6—C7	1.530 (3)
N1—H1B	0.94 (3)	C6—H6A	0.9900
N1—H1C	0.976 (17)	C6—H6B	0.9900
C1—C8	1.526 (3)	C7—C10	1.530 (3)
C1—C9	1.527 (3)	C7—C8	1.537 (3)
C1—C2	1.530 (3)	C7—H7A	1.0000
O2—C11	1.241 (3)	C8—H8A	0.9900
C2—C3	1.530 (3)	C8—H8B	0.9900
C2—H2B	0.9900	C9—H9A	0.9900
C2—H2A	0.9900	C9—H9B	0.9900
C3—C4	1.530 (3)	C10—H10A	0.9900
C3—C10	1.532 (3)	C10—H10B	0.9900
C3—H3A	1.0000	C11—C12	1.510 (3)
C4—C5	1.533 (3)	C12—H12A	0.9800
C4—H4A	0.9900	C12—H12B	0.9800
C4—H4B	0.9900	C12—H12C	0.9800
C5—C6	1.529 (3)		
			
C1—N1—H1A	110.6 (16)	C7—C6—H6A	109.7
C1—N1—H1B	109.1 (15)	C5—C6—H6B	109.7
H1A—N1—H1B	104 (2)	C7—C6—H6B	109.7
C1—N1—H1C	110.6 (14)	H6A—C6—H6B	108.2
H1A—N1—H1C	109 (2)	C6—C7—C10	109.25 (18)
H1B—N1—H1C	113 (2)	C6—C7—C8	109.50 (18)
N1—C1—C8	109.19 (17)	C10—C7—C8	109.58 (18)
N1—C1—C9	109.28 (17)	C6—C7—H7A	109.5
C8—C1—C9	109.84 (17)	C10—C7—H7A	109.5
N1—C1—C2	108.69 (17)	C8—C7—H7A	109.5
C8—C1—C2	109.66 (17)	C1—C8—C7	109.02 (17)
C9—C1—C2	110.15 (17)	C1—C8—H8A	109.9
C3—C2—C1	109.15 (17)	C7—C8—H8A	109.9
C3—C2—H2B	109.9	C1—C8—H8B	109.9
C1—C2—H2B	109.9	C7—C8—H8B	109.9
C3—C2—H2A	109.9	H8A—C8—H8B	108.3
C1—C2—H2A	109.9	C1—C9—C5	109.05 (18)
H2B—C2—H2A	108.3	C1—C9—H9A	109.9
C2—C3—C4	109.27 (19)	C5—C9—H9A	109.9
C2—C3—C10	109.36 (18)	C1—C9—H9B	109.9
C4—C3—C10	109.87 (19)	C5—C9—H9B	109.9
C2—C3—H3A	109.4	H9A—C9—H9B	108.3
C4—C3—H3A	109.4	C7—C10—C3	109.35 (18)
C10—C3—H3A	109.4	C7—C10—H10A	109.8
C3—C4—C5	109.68 (18)	C3—C10—H10A	109.8
C3—C4—H4A	109.7	C7—C10—H10B	109.8
C5—C4—H4A	109.7	C3—C10—H10B	109.8
C3—C4—H4B	109.7	H10A—C10—H10B	108.3
C5—C4—H4B	109.7	O2—C11—O1	125.0 (2)
H4A—C4—H4B	108.2	O2—C11—C12	118.0 (2)
C6—C5—C4	109.34 (19)	O1—C11—C12	117.1 (2)
C6—C5—C9	109.52 (18)	C11—C12—H12A	109.5
C4—C5—C9	109.03 (18)	C11—C12—H12B	109.5
C6—C5—H5A	109.6	H12A—C12—H12B	109.5
C4—C5—H5A	109.6	C11—C12—H12C	109.5
C9—C5—H5A	109.6	H12A—C12—H12C	109.5
C5—C6—C7	109.83 (18)	H12B—C12—H12C	109.5
C5—C6—H6A	109.7		
			
N1—C1—C2—C3	179.86 (18)	C9—C1—C8—C7	−60.8 (2)
C8—C1—C2—C3	−60.8 (2)	C2—C1—C8—C7	60.4 (2)
C9—C1—C2—C3	60.2 (2)	C6—C7—C8—C1	59.8 (2)
C1—C2—C3—C4	−59.9 (2)	C10—C7—C8—C1	−60.0 (2)
C1—C2—C3—C10	60.4 (2)	N1—C1—C9—C5	−179.49 (17)
C2—C3—C4—C5	60.6 (2)	C8—C1—C9—C5	60.7 (2)
C10—C3—C4—C5	−59.4 (2)	C2—C1—C9—C5	−60.1 (2)
C3—C4—C5—C6	59.2 (2)	C6—C5—C9—C1	−59.7 (2)
C3—C4—C5—C9	−60.5 (2)	C4—C5—C9—C1	59.9 (2)
C4—C5—C6—C7	−59.9 (2)	C6—C7—C10—C3	−60.0 (2)
C9—C5—C6—C7	59.5 (2)	C8—C7—C10—C3	59.9 (2)
C5—C6—C7—C10	60.5 (2)	C2—C3—C10—C7	−60.2 (2)
C5—C6—C7—C8	−59.5 (2)	C4—C3—C10—C7	59.8 (2)
N1—C1—C8—C7	179.39 (17)		

### Hydrogen-bond geometry (Å, °)

**Table:**

*D*—H···*A*	*D*—H	H···*A*	*D*···*A*	*D*—H···*A*
N1—H1A···O1	0.99 (4)	1.80 (4)	2.777 (3)	171 (3)
N1—H1B···O2^i^	0.94 (3)	1.83 (3)	2.758 (3)	170 (3)
N1—H1C···O1^ii^	0.97 (2)	1.82 (2)	2.786 (2)	171 (3)

Symmetry codes:  (i) −*x*+1/2, *y*−1/2, −*z*+3/2; (ii) −*x*+1/2, *y*+1/2, −*z*+3/2.

## References

[bb1] Atwood, J. L. & Barbour, L. J. (2003). *Cryst. Growth Des.* **3**, 3–8.

[bb2] Barbour, L. J. (2001). *J. Supramol. Chem.* **1**, 189–191.

[bb3] Bernstein, J., Davis, R. E., Shimoni, L. & Chang, N.-L. (1995). *Angew. Chem. Int. Ed. Engl.* **35**, 1555–1573.

[bb4] Betakova, T. (2007). *Curr. Pharm. Des.* **13**, 3231–3235. 10.2174/138161207782341295 18045172

[bb5] Bright, R. A., Medina, M. J., Xu, X. Y., Gilda, P. O., Wallis, T. R., Davis, X. H. M., Povinelli, L., Cox, N. J. & Klimov, A. I. (2005). *Lancet*, **366**, 1175–1181. 10.1016/S0140-6736(05)67338-2 16198766

[bb6] Bruker (2005). *APEX2* and *SAINT-NT* Bruker AXS Inc., Madison, Wisconsin, USA.

[bb7] Dolin, R., Reichman, R. C., Madore, H. P., Maynard, R., Lindon, P. M. & Weber-Jones, J. (1982). *N. Engl. J. Med.* **307**, 580–584. 10.1056/NEJM198209023071002 7050702

[bb8] He, Y.-H. & Wen, Y.-H. (2006). *Acta Cryst.* E**62**, o1312–o1313.

[bb9] Hoffmann, C. E. (1973). *Selective Inhibitors of Viral Functions*, edited by W. A. Carter, p. 199. Cleveland, USA: CRC Press.

[bb10] Mullica, D. F., Scott, T. G., Farmer, J. M. & Kautz, J. A. (1999). *J. Chem. Crystallogr.* **29**, 845–848.

[bb11] Orpen, A. G., Brammer, L., Allen, F. H., Kennard, O., Watson, D. G. & Taylor, R. (1989). *J. Chem. Soc. Dalton Trans.* pp. S1–83.

[bb12] Sheldrick, G. M. (2008). *Acta Cryst.* A**64**, 112–122. 10.1107/S0108767307043930 18156677

